# *Yersinia pestis* Requires Host Rab1b for Survival in Macrophages

**DOI:** 10.1371/journal.ppat.1005241

**Published:** 2015-10-23

**Authors:** Michael G. Connor, Amanda R. Pulsifer, Christopher T. Price, Yousef Abu Kwaik, Matthew B. Lawrenz

**Affiliations:** Department of Microbiology and Immunology and the Center for Predictive Medicine for Biodefense and Emerging Infectious Diseases, University of Louisville School of Medicine, Louisville, Kentucky, United States of America; Stanford University School of Medicine, UNITED STATES

## Abstract

*Yersinia pestis* is a facultative intracellular pathogen that causes the disease known as plague. During infection of macrophages *Y*. *pestis* actively evades the normal phagosomal maturation pathway to establish a replicative niche within the cell. However, the mechanisms used by *Y*. *pestis* to subvert killing by the macrophage are unknown. Host Rab GTPases are central mediators of vesicular trafficking and are commonly targeted by bacterial pathogens to alter phagosome maturation and killing by macrophages. Here we demonstrate for the first time that host Rab1b is required for *Y*. *pestis* to effectively evade killing by macrophages. We also show that Rab1b is specifically recruited to the *Yersinia* containing vacuole (YCV) and that *Y*. *pestis* is unable to subvert YCV acidification when Rab1b expression is knocked down in macrophages. Furthermore, Rab1b knockdown also altered the frequency of association between the YCV with the lysosomal marker Lamp1, suggesting that Rab1b recruitment to the YCV directly inhibits phagosome maturation. Finally, we show that Rab1b knockdown also impacts the pH of the *Legionella pneumophila* containing vacuole, another pathogen that recruits Rab1b to its vacuole. Together these data identify a novel role for Rab1b in the subversion of phagosome maturation by intracellular pathogens and suggest that recruitment of Rab1b to the pathogen containing vacuole may be a conserved mechanism to control vacuole pH.

## Author Summary

*Yersinia pestis* is the bacterial agent that causes the human disease known as plague. While often considered a historic disease, *Y*. *pestis* is endemic in rodent populations on several continents and the World Health Organization considers plague to be a reemerging disease. Much of the success of this pathogen comes from its ability to evade clearance by the innate immune system of its host. One weapon in the *Y*. *pestis* arsenal is its ability to resist killing when engulfed by macrophages. Upon invasion of macrophages, *Y*. *pestis* actively manipulates the cell to generate a protective vacuolar compartment, called the *Yersinia* containing vacuole (YCV) that allows the bacterium to evade the normal pathogen killing mechanisms of the macrophage. Here we demonstrate that the host protein Rab1b is recruited to the YCV and is required for *Y*. *pestis* to inhibit both the acidification and normal maturation of the phagosome to establish a protective niche within the cell. Rab1b is the first protein, either from the host or *Y*. *pestis*, shown to contribute to the biogenesis of the YCV. Furthermore, our data suggest a previously unknown impact of Rab1b recruitment in the phagosome maturation pathway.

## Introduction

*Yersinia pestis* is a facultative intracellular pathogen and causative agent of the disease known as plague. There have been three human plague pandemics in history; the most notable being the Black Death in the 14^th^ century [1, 2]. *Y*. *pestis* can infect humans either through the bite of an infected flea or inhalation of contaminated aerosols. Flea inoculation can lead to the development of bubonic plague, a form of plague highlighted by bacterial dissemination to, and replication within, lymph nodes [1]. Inhalation of *Y*. *pestis* contaminated aerosols can result in rapid colonization of the lungs and development of pneumonic plague [1]. Both forms of plague are associated with acute disease progression and high mortality rates in the absence of timely antibiotic treatment. Furthermore, the potential for person-to-person transmission and use as a biological weapon in the absence of a vaccine highlights the risks associated with this pathogen [3].

During its natural life cycle, *Y*. *pestis* cycles between two different hosts, the mammal and the flea. The bacterium requires different virulence factors to colonize each host, and coordinates the expression of these factors accordingly [1]. *Y*. *pestis* has several well characterized antiphagocytic mammalian virulence factors, such as the Ysc type three secretion system (T3SS), secreted Yop effectors and the Caf1 capsule [1]. However, these virulence factors are down regulated in the flea vector and at the time of initial colonization of the mammalian host [1]. During this transitional period, *Y*. *pestis* is highly susceptible to phagocytosis by macrophages and neutrophils [4, 5]. Initial colonization of *Y*. *pestis* induces a rapid and early influx of neutrophils to the site of infection [4, 6]. Upon phagocytosis by neutrophils, *Y*. *pestis* is readily killed by these professional phagocytes [7–9]. However, *Y*. *pestis* has demonstrated an increased ability to survive phagocytosis by monocytes and macrophages [4, 5, 10–12]. Upon entry into the macrophage, *Y*. *pestis* actively circumvents the natural maturation of the phagolysosome by remodeling the phagosome into a hospitable replicative niche called the *Yersinia* containing vacuole (YCV) [11–15]. *In vitro* studies have highlighted three key characteristics of the biogenesis of the YCV. First, *Y*. *pestis* is able to actively inhibit the normal acidification of the phagosome and maintain a pH between 6.5–7.5 within the YCV throughout the course of intracellular infection [12]. Second, a significant portion of YCVs appear to become autophagosomes, which is highlighted by colocalization with LC3-II and the presence of double membranes surrounding the bacteria [12, 16]. While the contribution of autophagy to intracellular survival is unclear, data indicates that autophagy contributes to the metabolism of intracellular bacteria [16, 17]. Finally, approximately eight hours after phagocytosis, the tight fitting vacuolar membrane of the YCV begins to expand in size to form a spacious vacuolar compartment that can be observed by both light and electron microscopy [5, 12, 13, 18]. Bacterial replication within the YCV usually coincides with spacious vacuole formation. Importantly, while the fate of *Y*. *pestis* in the macrophage has been characterized, the mechanisms used to generate the YCV and avoid macrophage killing have not been defined.

The ability of *Y*. *pestis* to survive within macrophages also appears to impact virulence of the bacterium. *In vivo*, intracellular *Y*. *pestis* are recovered from macrophages isolated from both infected nonhuman primates and rodents, but rarely from neutrophils isolated from the same animals [7, 19, 20]. Ye and colleagues further showed lower bacterial burdens in transgenic MaFIA mice selectively depleted of macrophage/dendritic cell populations, suggesting that macrophages are required to establish acute infection [21]. *Y*. *pestis phoPQ* mutants, which are defective for intracellular survival, are also attenuated during subcutaneous infection of BALB/c (75-fold change in LD_50_) and Swiss Webster mice (no change in LD_50_ but a significant delay in time to death for mutant infected animals) [22, 23]. Moreover, macrophages isolated from canines, a species that are relatively resistant to plague [24], are significantly more capable in killing *Y*. *pestis* than macrophages isolated from laboratory mice, a species highly susceptible to plague, suggesting that the ability of macrophages to kill *Y*. *pestis* may contribute to resistance to infection [18]. Together, these data highlight the importance of *Y*. *pestis* survival within the macrophage during pathogenesis.

Rab GTPases are the largest member of the Ras Superfamily of small guanine triphosphatases and are central mediators of vesicle trafficking within eukaryotic cells [25, 26]. These GTPases mediate vesicle trafficking by cycling through active GTP-bound and inactive GDP-bound conformations [25, 26]. When bound to GTP, the Rab protein integrates into specific vesicle membranes to mediate the trafficking of that vesicle through interactions with other trafficking proteins. Hydrolysis of the bound GTP to GDP results in extraction of the Rab from the membrane. While approximately 60 different Rab proteins have been identified, the contributions of only a few Rabs to specific vesicle trafficking steps have been experimentally described. For example, Rab5, Rab7, and Rab9 have been well studied as key mediators of important steps in the phagosome maturation process [27–32]. Rab5 is recruited to the early endosome/phagosome and is required for phagocytosis [27–32]. Following phagocytosis, Rab5 disassociates from the early endosome and Rab7 is recruited to the endosome to facilitate recruitment of Rab9 and subsequent fusion with the lysosome [27–32]. A single disruption in the recruitment of a Rab protein to the maturing vesicle can stall and even terminate trafficking of that particular endocytic vesicle to its intended destination.

Due to the central role of Rab proteins for endosome sorting and phagosome maturation, many intracellular pathogens target Rab proteins to subvert these processes (see [25] for review). A classic example of Rab manipulation is seen in *Mycobacterium* infection of macrophages. *M*. *avium* and *M*. *tuberculosis* alter the normal distribution of Rab5 and Rab7 on their vacuole—retention of Rab5 and exclusion of Rab7 –to inhibit phagosomal fusion with the lysosome and subsequent killing of the bacteria [33–38]. More recently, Rab1 has emerged as a common target required for the intracellular survival of many pathogens [37, 39–49]. Rab1 has two isoforms, Rab1a and Rab1b, which share 92% amino acid similarity and are thought to be functionally redundant [50, 51]. Both isoforms have been shown to be involved in ER-to-Golgi trafficking [43, 52]. More recently Rab1a has been associated with proper endosome sorting during receptor mediated endocytosis and Rab1b has also been linked to autophagosome formation [44, 53–56]. Several pathogen containing vacuoles (PCVs) have been shown to associate with Rab1, and this recruitment is essential for subsequent survival of the pathogens contained within the PCV [39–47, 57]. *Coxiella burnetii* requires Rab1 for the *Coxiella* replicative vacuole (CRV) to expand in both Chinese hamster ovary (CHO) and RAW264.7 macrophage cells [39]. This expansion is significantly hindered in the presence of a GTP restricted form of Rab1 [39]. Similarly, *Anaplasma phagocytophilum* also recruits Rab1 directly to the *Anaplasma* containing vacuole (APV) and it has been speculated that recruitment of Rab1 to the APV allows the bacteria to hijack endocytic trafficking [43]. Perhaps the best studied subversion of Rab1 by a pathogen comes from *Legionella pneumophila*. Rab1 has been shown to accumulate on the *L*. *pneumophila* containing vacuole (LCV) as early as 10 min after bacterial uptake and Rab1 knockdown has been shown to inhibit *L*. *pneumophila* intracellular replication [40, 42, 46]. Furthermore, several *L*. *pneumophila* secreted effectors have been identified that specifically target and modify Rab1 to alter its localization [40, 42, 46, 47, 57–62]. In contrast to the requirement of Rab1 for the survival of these intracellular pathogens that exist within vacuoles, *Shigella flexneri*, which replicates in the host cytoplasm, is hindered by Rab1 [41]. Inactivation of Rab1 by *S*. *flexneri* is critical for bacterial survival and is mediated by the VirA/EspG secreted effector family [41]. Together, these studies suggest a distinct role for host Rab1 GTPases for intracellular survival of pathogens that replicate within vacuolar compartments.

Since Rab1 appears to be targeted by several pathogens that reside within vacuoles in order to survive intracellularly, we investigated the role of Rab1 in the survival of *Y*. *pestis* within macrophages. We demonstrate that siRNA knockdown of Rab1b in macrophages infected with *Y*. *pestis* significantly increases YCV acidification and association with the lysosomal marker Lamp1, resulting in decreased intracellular survival of *Y*. *pestis*. Furthermore, we show Rab1b is recruited to the YCV, suggesting a direct interaction with Rab1b is required for proper YCV maturation. Importantly, Rab1b is the first host protein to be identified that is required by *Y*. *pestis* to alter phagosome maturation and YCV acidification and impact the ability of this pathogen to survive within the eukaryotic cell. Finally, we also demonstrate for the first time that Rab1b recruitment to the *L*. *pneumophila* containing vacuole also impacts vacuole pH, suggesting a conserved mechanism for the recruitment of Rab1b to pathogen containing vacuoles.

## Results

### Rab1b is required for *Y*. *pestis* survival in macrophages

**Fig 1:**
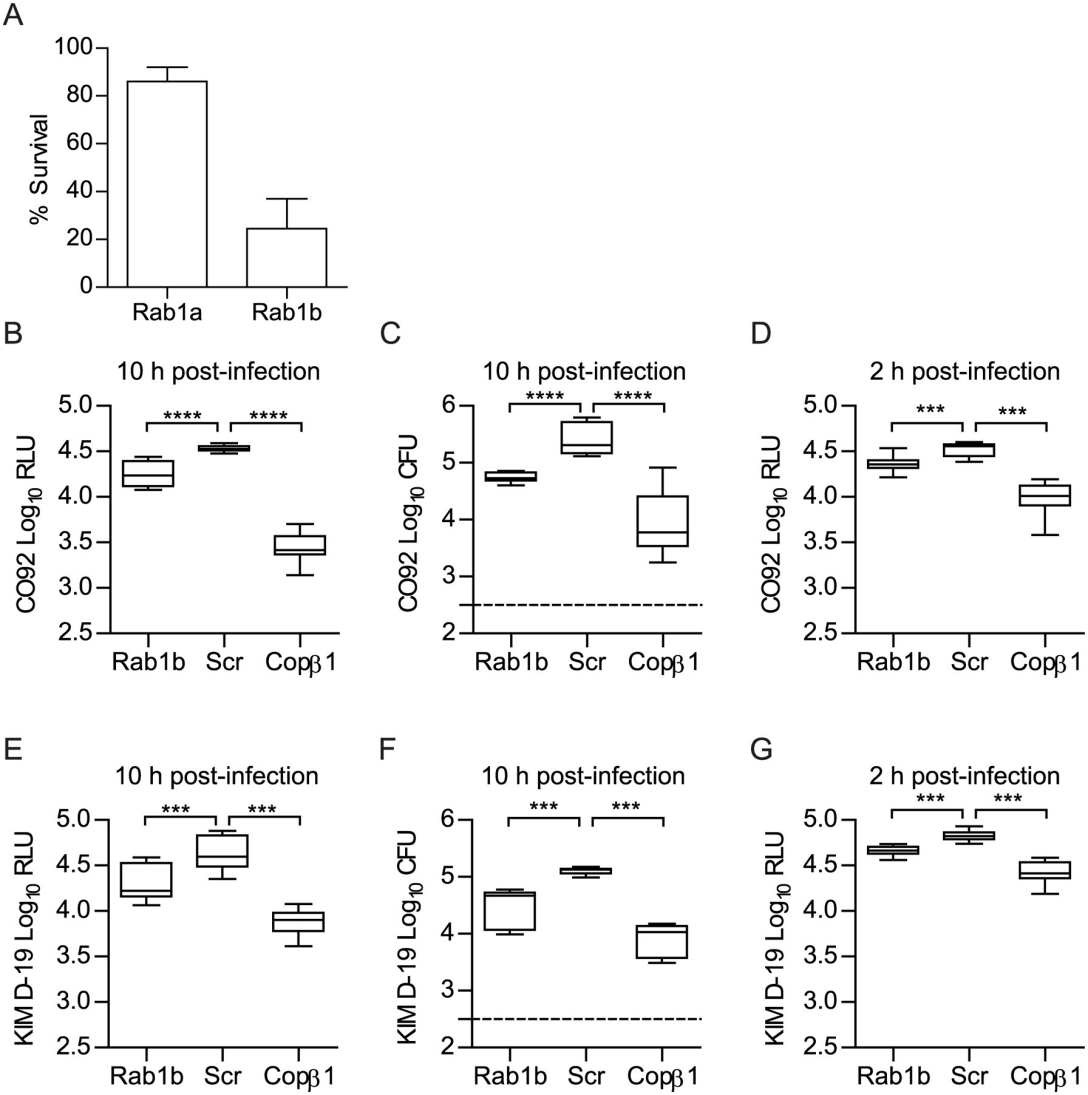
Rab1b knockdown inhibits the survival of *Y*. *pestis* within macrophages. RAW264.7 macrophages were reverse transfected with Rab1a, Rab1b, scrambled (Scr), or Copβ1 siRNA. 48 h after transfection cells were infected with *Y*. *pestis* (MOI 10). (A) Percent survival of intracellular CO92 pCD1^(-)^ Lux_PtolC_ in Rab1a or Rab1b siRNA treated macrophages as compared to Scr siRNA treated macrophages. (B) Bioluminescence of intracellular bacteria from macrophages infected for 10 h with *Y*. *pestis* CO92 pCD1^(-)^ Lux_PtolC_. (C) Conventional enumeration of intracellular bacteria from macrophages infected for 10 h with *Y*. *pestis* CO92 pCD1^(-)^ Lux_PtolC_. (D) Bioluminescence of intracellular bacteria from macrophages infected for 2 h with *Y*. *pestis* CO92 pCD1^(-)^ Lux_PtolC_. (E) Bioluminescence of intracellular bacteria from macrophages infected for 10 h with *Y*. *pestis* KIM D-19 Lux_PtolC_. (F) Conventional enumeration of intracellular bacteria from macrophages infected for 10 h with *Y*. *pestis* KIM D-19 Lux_PtolC_. (G) Bioluminescence of intracellular bacteria from macrophages infected for 2 h with *Y*. *pestis* KIM D-19 Lux_PtolC_. The limit of detection for conventional enumeration is denoted by the dotted line. RLU = Relative Light Units; CFU = Colony Forming Units. *** = p<0.001, **** = p<0.0001.

Since *Y*. *pestis* exists within a vacuolar compartment within macrophages [5, 11, 14], and Rab1 has been linked to survival of several other intracellular pathogens that exist within vacuoles [39, 40, 43–46, 48], we sought to determine if Rab1 is required for *Y*. *pestis* intracellular survival. Toward this goal, we initially screened whether either isoform of Rab1 is required for *Y*. *pestis* to survive in macrophages. RAW264.7 macrophages were transfected with either Rab1a or Rab1b specific siRNAs (pool of 3 siRNAs targeting each gene). 48 h after transfection, macrophages were infected with *Y*. *pestis* CO92 pCD1^(-)^ Lux_PtolC_, which contains a bioluminescent bioreporter to monitor *Y*. *pestis* numbers [63]. Extracellular bacteria were killed with gentamicin, and intracellular bacterial survival was monitored via bioluminescent signal (Fig 1A). While no change in *Y*. *pestis* bioluminescence was observed in Rab1a siRNA treated cells compared to scrambled siRNA treated controls, we observed a significant decrease in bioluminescence in Rab1b siRNA treated cells, indicating that Rab1b, but not Rab1a, is required for *Y*. *pestis* survival within macrophages.

To confirm Rab1b is required for *Y*. *pestis* intracellular survival, RAW264.7 macrophages were transfected with a single Rab1b siRNA optimized for Rab1b knockdown and cell viability (S1 Fig) and infected with *Y*. *pestis* CO92 pCD1^(-)^ Lux_PtolC_ 48 h post-transfection. As a positive control, we also infected macrophages transfected with Copβ1 siRNA. Copβ1 is a component of the cotamer complex and has been shown to alter both invasion and survival of other intracellular pathogens [64, 65]. As expected, Copβ1 knockdown resulted in a significant decrease in intracellular *Y*. *pestis* CO92 pCD1^(-)^ Lux_PtolC_ bioluminescence at 10 h post-infection as compared to scramble siRNA treated cells (Fig 1B; p≤0.0001). Rab1b knockdown also resulted in a significant decrease in bioluminescent signal; *Y*. *pestis* CO92 pCD1^(-)^ Lux_PtolC_ bioluminescence was ~50% less in Rab1b siRNA treated cells (Fig 1B; p≤0.0001). To confirm that *Y*. *pestis* CO92 pCD1^(-)^ Lux_PtolC_ bioluminescence accurately represents viable intracellular bacteria, cells were lysed and bacterial numbers were determined by conventional serial dilution enumeration (Fig 1C). Conventional enumeration supported our bioluminescent data and demonstrated a significant decrease in viable intracellular colony forming units (CFU) in Rab1b siRNA treated cells (p≤0.001). No differences in survival were observed if bacteria were grown at 37°C prior to infection (S2 Fig). Importantly, the direct correlation between bioluminescent signal and bacterial enumeration support the use of bioluminescent data to monitor intracellular *Y*. *pestis* numbers.

To confirm that the pCD1 encoded Ysc type three secretion system (T3SS) does not impact Rab1b mediated *Y*. *pestis* survival, Rab1b transfected cells were also infected with *Y*. *pestis* KIM D-19 Lux_PtolC_, which contains the pCD1 plasmid and the Ysc T3SS, and bacterial survival was monitored by bioluminescence and conventional bacterial enumeration (Fig 1E and 1F). As observed for *Y*. *pestis* CO92 pCD1^(-)^ Lux_PtolC_, we observed an ~50% decrease in *Y*. *pestis* KIM D-19 Lux_PtolC_ survival in Rab1b siRNA treated cells (p≤0.001). We also monitored *Y*. *pestis* intracellular bioluminescence temporally over the course of the infection to determine how early during infection *Y*. *pestis* intracellular survival was impacted by Rab1b knockdown. This analysis revealed that intracellular bacterial numbers for both strains were significantly decreased in Rab1b treated cells as early as 2 h post-infection, which is the earliest time point we can monitor after gentamicin removal (Fig 1D and 1G; p≤0.001). Finally, to determine if the Rab1b impact on intracellular survival is conserved in the *Yersinia* genus, transfected macrophages were infected with *Y*. *pseudotuberculosis* and *Y*. *enterocolitica*. As observed for *Y*. *pestis*, both enteric species were attenuated in survival when Rab1b was knocked down (S3 Fig). Together these data demonstrate that Rab1b is required for *Yersinia* intracellular survival, which is independent of the Ysc T3SS, and bacterial survival is impacted by Rab1b very early during the infection process.

### Rab1b is not required for *Y*. *pestis* invasion of macrophages

**Fig 2:**
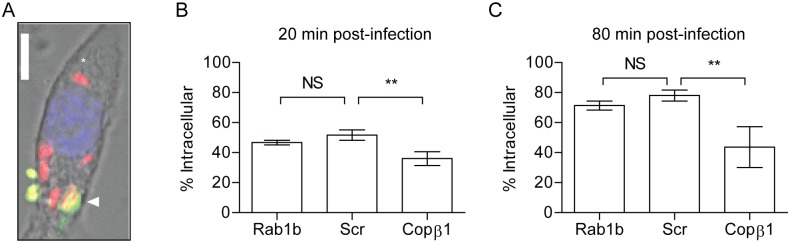
Rab1b knockdown does not impact *Y*. *pestis* invasion of macrophages. RAW264.7 macrophages were reverse transfected with Rab1b, scrambled (Scr), or Copβ1 siRNA. 48 h after transfection cells were infected with *Y*. *pestis* CO92 pCD1^(-)^pGEN-*P*_*EM7*_::DsRED (MOI 7.5). 20 or 80 min post-infection cells and bacteria were fixed with paraformaldehyde and extracellular bacteria were stained by indirect immunofluorescence with anti-*Y*. *pestis* antibody. (A) Representative image showing differential staining of intracellular (red) and extracellular (green or yellow) bacteria. Scale bar is 5μm. Asterisk denotes intracellular *Y*. *pestis*. (B and C) Percentage of intracellular bacteria calculated at 20 and 80 min post-infection, respectively. ** = p<0.01, ns = not significant.

We observed a difference in *Y*. *pestis* intracellular numbers in Rab1b siRNA treated cells within 2 h of macrophage infection (Fig 1D and 1G). The difference in recovered bacteria at this early time point could be due to an inability of *Y*. *pestis* to avoid phaogolysomal killing in the absence of Rab1b. However, Rab1b may also be required for efficient phagocytosis and the difference in *Y*. *pestis* numbers at 2 h post-infection could be a result of less bacteria gaining entry into the macrophages prior to gentamicin treatment. Because phagolysosome fusion and bacterial killing can occur within 120 minutes of phagocytosis [25, 27], we could not rely on the conventional gentamicin protection assay, which requires a 1 h incubation period, to differentiate between invasion and bacterial killing in Rab1b siRNA treated cells. Therefore, we used a differential staining procedure to specifically label extracellular *Y*. *pestis* and determine if Rab1b knockdown impacted *Y*. *pestis* invasion of macrophages by confocal microscopy. Rab1b siRNA transfected RAW264.7 macrophages were infected with *Y*. *pestis* CO92 pCD1^(-)^ pGEN-*P*_*EM7*_::DsRED [66], which constitutively expresses the DsRED fluorescent protein. At 20 and 80 min post-infection, cells and total bacteria were fixed with paraformaldehyde. Extracellular bacteria were then specifically labeled with anti-*Y*. *pestis* polyclonal antibody and Alexa Fluor 488 anti-rabbit secondary antibody (Fig 2A). As a positive control, macrophages were treated with Copβ1 siRNA, which has been previously shown to be required for efficient phagocytosis [64]. As expected, cells treated with Copβ1 had significantly less intracellular *Y*. *pestis* than scrambled siRNA treated macrophages at both 20 and 80 min post-infection (Fig 2B and 2C; p≤0.001). Conversely, we observed no difference in the proportion of intracellular *Y*. *pestis* in Rab1b siRNA treated cells compared to scrambled siRNA treated cells. These data demonstrate that Rab1b is not required for phagocytosis of *Y*. *pestis* and suggest that the differences in intracellular bacterial numbers in Rab1b siRNA treated cells is due to a decreased ability of *Y*. *pestis* to avoid macrophage killing in the absence of Rab1b.

### Rab1b is required for *Y*. *pestis* to avoid YCV acidification

**Fig 3:**
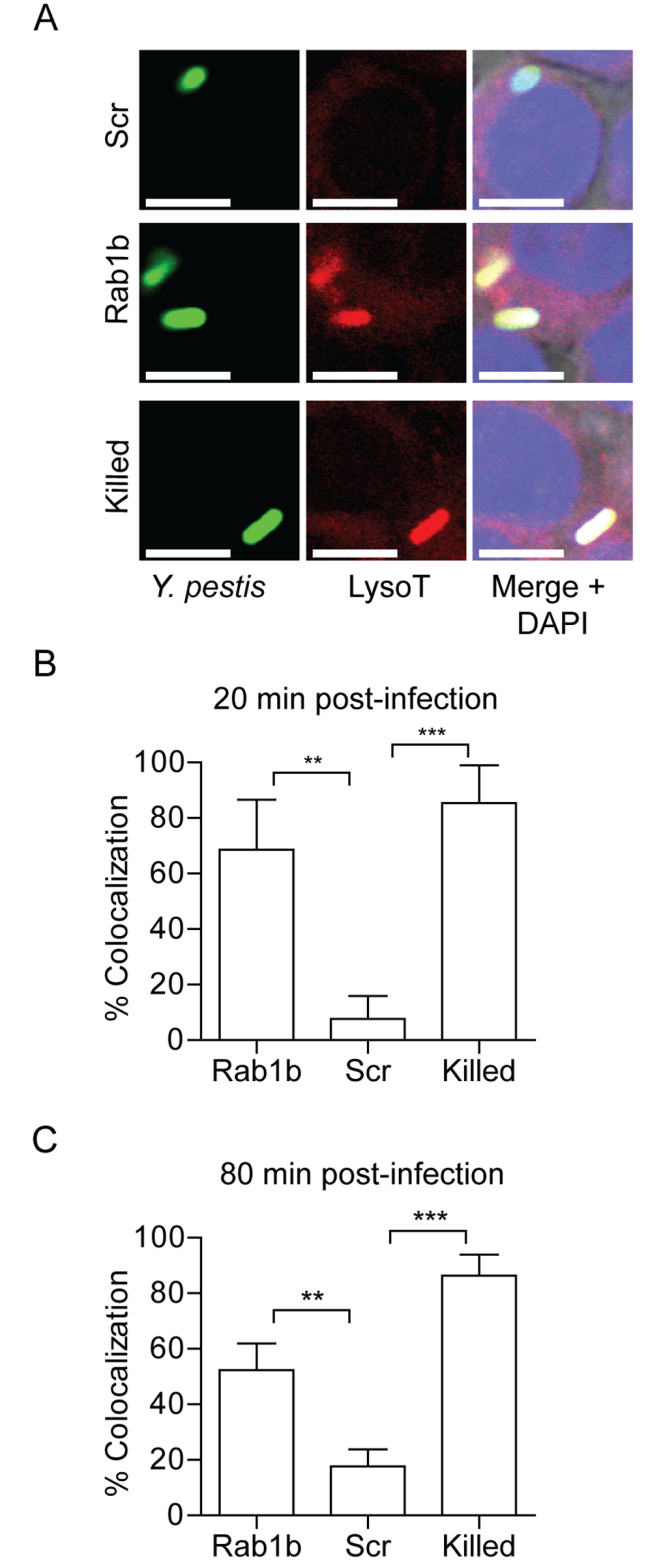
Rab1b knockdown alters YCV acidification. RAW264.7 macrophage cells were reverse transfected with scrambled (Scr), Rab1b or Copβ1 siRNA. 48 h after transfection cells were incubated with Lysotracker Red DND-99 for 1 h and then infected with live or paraformaldehyde-killed *Y*. *pestis* CO92 pCD1^(-)^ pGEN222 expressing EGFP (MOI 7.5). Colocalization of Lysotracker Red DND-99 and *Y*. *pestis* CO92 pCD1^(-)^ pGEN222 was determined by confocal microscopy. (A) Representative images showing colocalization of Lysotracker Red DND-99 and *Y*. *pestis*. Scale bar is 5μm. (B) Percent of YCVs that colocalized with Lysotracker Red DND-99 at 20 min post-infection. (C) Percent of YCVs that colocalized with Lysotracker Red DND-99 at 80 min post-infection. ** = p<0.01, *** = p<0.001.

A hallmark characteristic of *Y*. *pestis* infection of the macrophage is that the bacterium is able to rapidly subvert normal acidification of the YCV [12]. Because acidification is one of the earliest steps in phagosome maturation and is required for both efficient lysosomal fusion and degradation of phagolysosomal contents [27], we next investigated whether Rab1b is required for *Y*. *pestis* to avoid YCV acidification. RAW264.7 macrophages were transfected with Rab1b siRNA and then treated with Lysotracker Red DND-99 prior to infection with *Y*. *pestis* CO92 pCD1^(-)^ pGEN222, which constitutively expresses EGFP. Lysotracker Red DND-99 fluorescence is pH dependent (fluoresces below pH 5.5), and therefore, allows for identification of acidified vacuoles. As *Y*. *pestis* inhibition of YCV acidification is an active process, untransfected cells were infected with paraformaldehyde killed *Y*. *pestis* CO92 pCD1^(-)^ pGEN222 to serve as a positive control for YCV acidification. As previously reported for untransfected macrophages [12], *Y*. *pestis* CO92 pCD1^(-)^ pGEN222 efficiently avoided YCV acidification in scramble siRNA treated macrophages, with <25% of *Y*. *pestis* found within acidified vacuoles by 80 min post-infection (Fig 3). This was significantly lower than paraformaldehyde killed *Y*. *pestis*, which were already within acidified vacuoles >80% of the time by 20 min post-infection (p≤0.01). The ability of *Y*. *pestis* to inhibit YCV acidification was greatly attenuated in Rab1b knocked down cells, where ~70% of the bacteria were observed within acidified vacuoles within 20 min post-infection (p≤0.01). Furthermore, *Y*. *pestis* remained within acidified vacuoles in Rab1b siRNA treated macrophages at 80 min post-infection. These data demonstrate that *Y*. *pestis* requires the host Rab1b GTPase to inhibit or avoid YCV acidification.

### Rab1b is necessary for *Y*. *pestis* to avoid fusion with the lysosome

**Fig 4:**
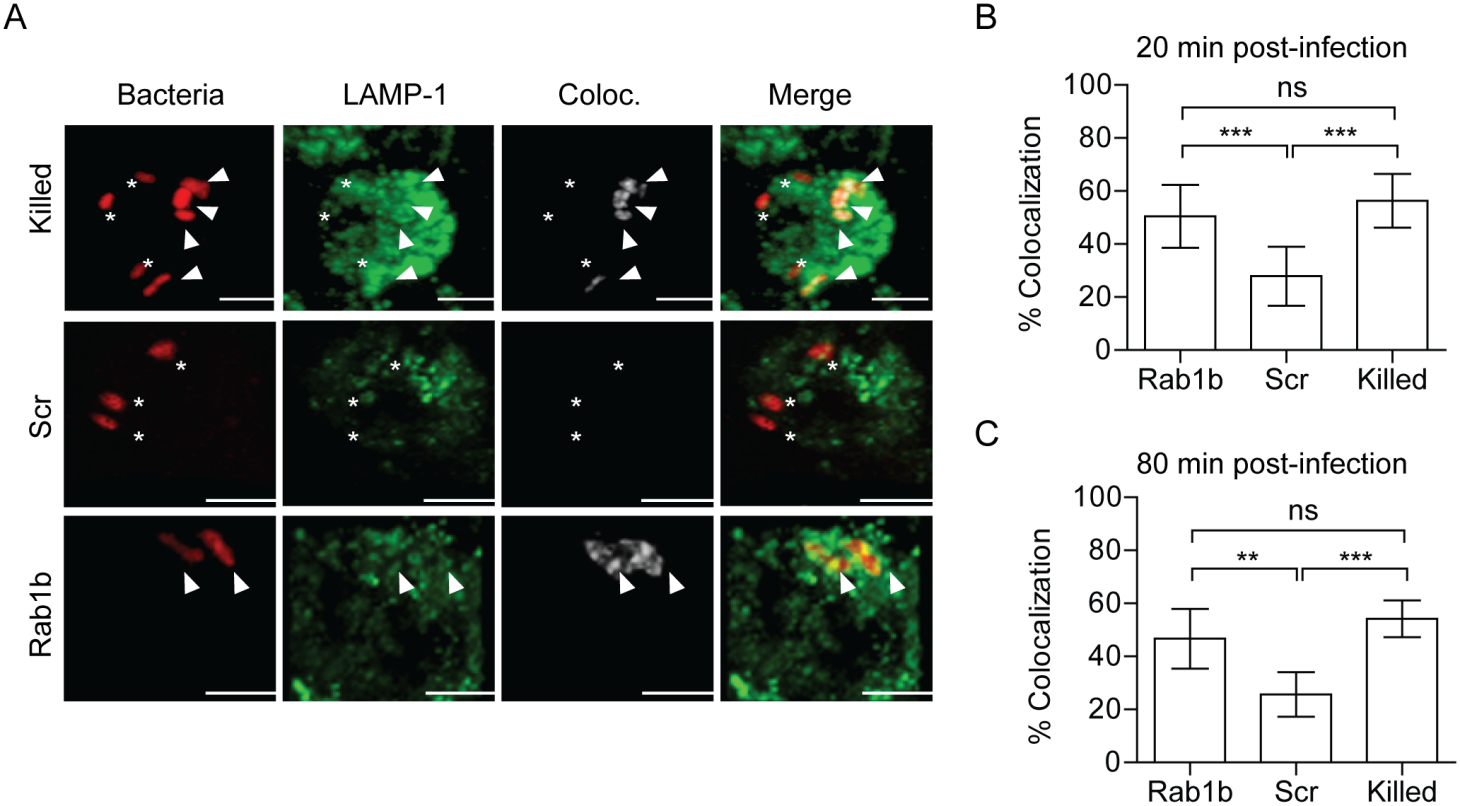
Rab1b knockdown increases YCV association with Lamp1. RAW264.7 macrophage cells were reverse transfected with either scrambled (Scr) or Rab1b siRNA. 48 h after transfection cells were infected with live or paraformaldehyde-killed *Y*. *pestis* CO92 pCD1^(-)^ pGEN-EM7::DsRED (MOI 3). Cells were stained for Lamp1 and colocalization was determined by confocal microscopy. (A) Representative images showing bacterial colocalization with Lamp1 at 20 min post-infection. Colocalization channel was defined using Imaris software. Asterisks denote bacteria not colocalized with Lamp1; arrowheads denote bacteria colocalized with Lamp1. Scale bar is 5μm. (B) Percent of YCVs that colocalized with Lamp1 at 20 min post-infection. (C) Percent of YCVs that colocalized with Lamp1 at 80 min post-infection. ** = p<0.01, *** = p<0.001.

Acidification of the phagosome precedes or coincides with fusion to lysosomes and degradation of foreign particles such as bacteria [27]. As Rab1b knockdown resulted in increased acidification of the YCV, we next determined if Rab1b is required for *Y*. *pestis* to avoid fusion with lysosomes. RAW264.7 macrophages were transfected with Rab1b siRNA and infected with live or paraformaldehyde killed *Y*. *pestis* CO92 pCD1^(-)^ pGEN-*P*_*EM7*_::DsRED. At 20 and 80 min post-infection, cells were washed, fixed with paraformaldehyde, and stained with anti-Lamp1 antibody, a marker for lysosomal fusion (Fig 4A). In scrambled siRNA treated cells, we observed minimal association of live *Y*. *pestis* with Lamp1 (<25%) at 20 and 80 min post-infection, indicating limited association between the YCV and lysosomes at these time points (Fig 4B and 4C). As observed for YCV acidification, there was a significant increase in the association between Lamp1 and paraformaldehyde killed *Y*. *pestis* (>60%), supporting an active avoidance of lysosomal fusion by *Y*. *pestis* during macrophage infection (Fig 4B and 4C; p≤0.001). Rab1b knockdown also significantly altered Lamp1 association with the YCV compared to scramble siRNA (Fig 4B and 4C; p≤0.001 and p≤0.01, respectively). At 20 min post-infection, Lamp1 associated with ~55% of YCVs in Rab1b siRNA treated cells, and was maintained at this elevated level at 80 min post-infection. These data indicate that Rab1b is required not only for *Y*. *pestis* to inhibit YCV acidification but also to avoid lysosomal fusion. Importantly, the ~2-fold increase in association with Lamp1 directly correlates to a similar 2-fold decrease in *Y*. *pestis* survival in Rab1b siRNA treated macrophages (Fig 1).

### Rab1b is not required for early *Y*. *pestis* association with LC3

**Fig 5:**
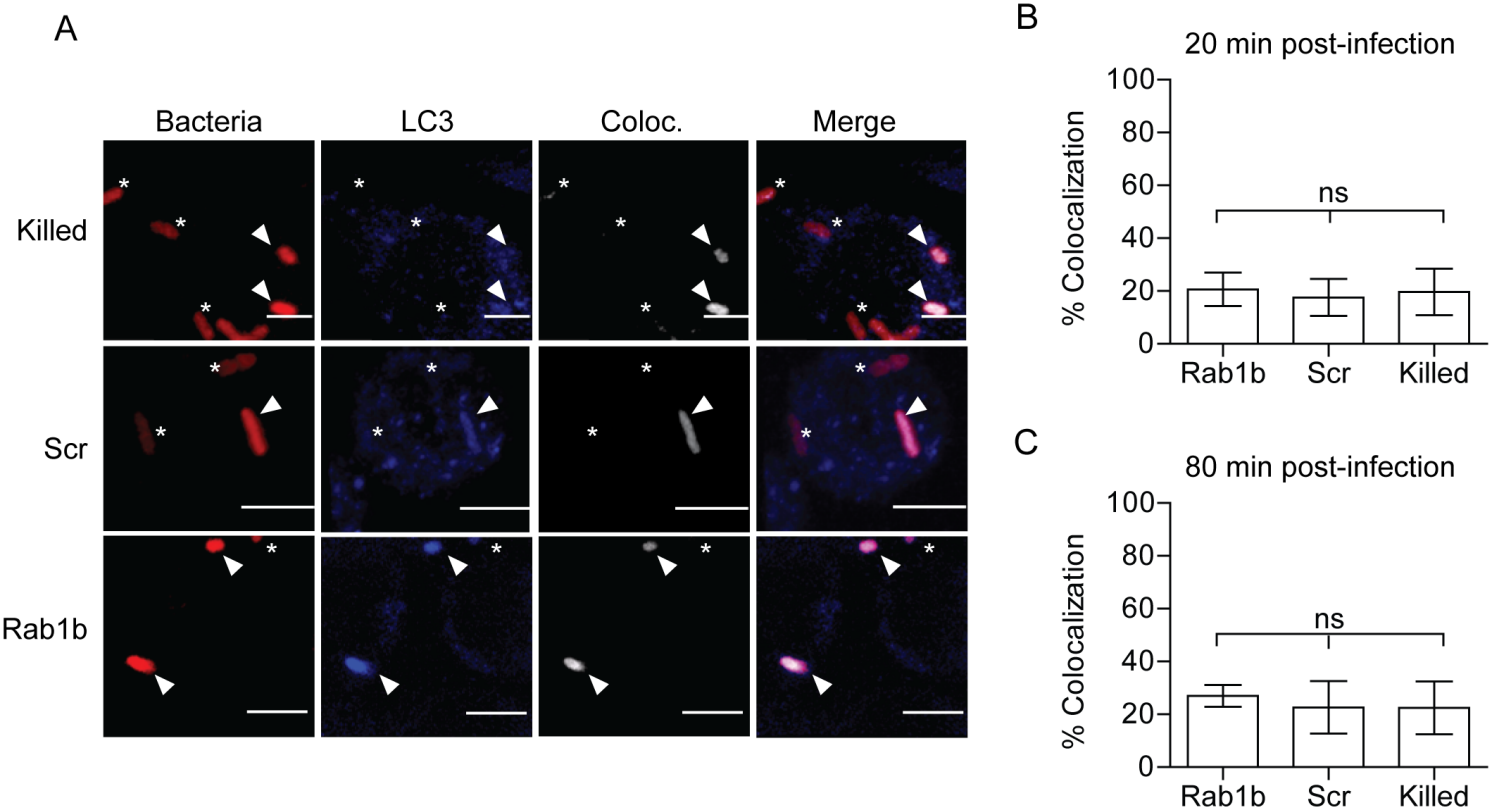
Rab1b knockdown does not affect YCV association with LC3. RAW264.7 macrophage cells were reverse transfected with either scrambled (Scr) or Rab1b siRNA. 48 h after transfection cells were infected with live or paraformaldehyde killed *Y*. *pestis* CO92 pCD1^(-)^ pGEN-*P*_*EM7*_::DsRED (MOI 7.5). Cells were stained for LC3 and colocalization was determined by confocal microscopy. (A) Representative images showing bacterial colocalization with LC3 at 20 min post infection. The colocalization channel was defined using Imaris software. Asterisks denote bacteria not colocalized with LC3; arrowheads denote bacteria colocalized with LC3. Scale bar is 5μm. (B) Percent of YCVs that colocalized with LC3 at 20 min post-infection. (C) Percent of YCVs that colocalized with LC3 at 80 min post-infection. ns = not significant.

Autophagy has been linked to both *Y*. *pestis* and *Y*. *pseudotuberculosis* intracellular infection and may be required for sustained bacterial metabolism within cells [12, 16]. Furthermore, studies have shown a recruitment of LC3, a marker for autophagosomes, to the YCV during *Y*. *pseudotuberculosis* infection of HeLa cells and BMDMs [16, 17]. Recently, Huang and colleagues demonstrated a potential role for Rab1b in autophagy and intracellular survival of *Salmonella enterica Typhimurium* [44]. Given the link of Rab1b to autophagy and autophagy to *Yersinia* intracellular infection, we next investigated if knockdown of Rab1b impacted early association of LC3 to the YCV during macrophage infection. RAW264.7 macrophages were transfected with Rab1b siRNA and infected with live or paraformaldehyde killed *Y*. *pestis* CO92 pCD1^(-)^ pGEN-*P*_*EM7*_::DsRED. 20 and 80 min post-infection cells were washed, fixed with paraformaldehyde, and stained with anti-LC3 antibody (Fig 5A). In contrast to reported infection of epithelial cells with *Y*. *pseudotuberculosis* [17], we observed a very low incidence in the association between live or killed *Y*. *pestis* with LC3 during early stages of macrophage infection (Fig 5B and 5C) and this association was not significantly altered in Rab1b siRNA treated cells (~20% association in all samples). These data support previous data that LC3 association with the YCV is lower in macrophages than epithelial cells [16, 17] and demonstrate that Rab1b knockdown does not alter YCV-LC3 association during the early stages of *Y*. *pestis* infection when we observe changes in YCV maturation and intracellular survival of the bacteria.

### Rab1b is recruited to the YCV during macrophage infection

**Fig 6:**
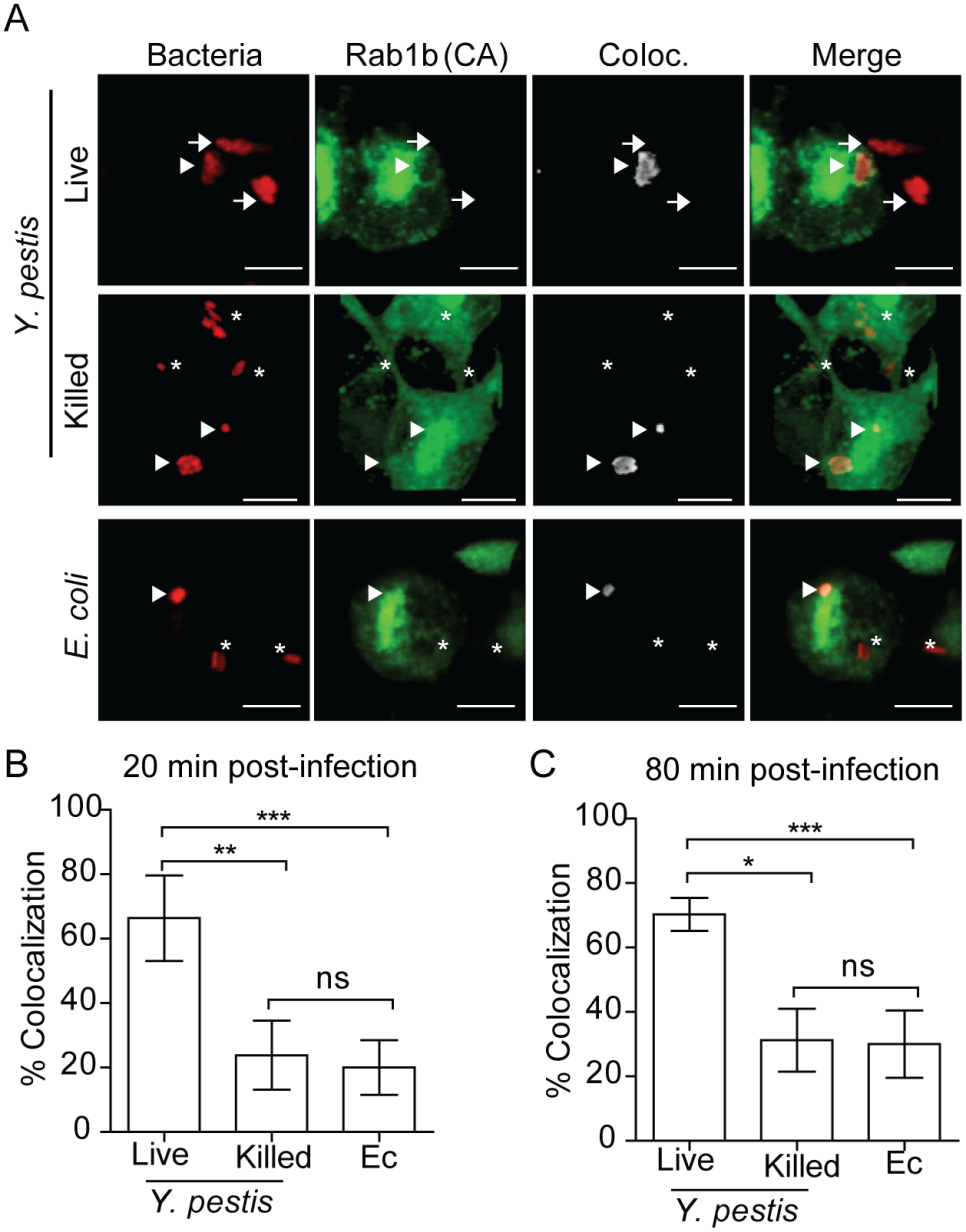
Rab1b is recruited to the YCV. RAW264.7 macrophages were transiently transfected with pEGFP-Rab1B(CA). 24 h after transfection cells were infected with either live or paraformaldehyde killed *Y*. *pestis* pMCherry (MOI 7.5) or *E*. *coli* pMCherry (MOI 20). Colocalization of EGFP-Rab1b(CA) and bacteria was determined by confocal microscopy. (A) Representative images showing bacterial colocalization with EGFP-Rab1b(CA). Colocalization channel was defined using Imaris software. Asterisks denote bacteria not colocalized with EGFP-Rab1b(CA); arrowheads denote bacteria colocalized with EGFP-Rab1b(CA); arrows denote bacteria in untransfected cells. Scale bar is 5μm. (B and C) Percent of bacteria colocalized with EGFP-Rab1B(CA) at 20 and 80 min post-infection. * = p<0.05; ** = p<0.01; *** = p<0.001.

Rab GTPases mediate vesicular trafficking through direct interactions with vesicle membranes (see [25, 26] for review). Therefore, we next sought to determine whether Rab1b is recruited to the YCV during *Y*. *pestis* infection. Because Rab interactions with membranes are transient, we transfected RAW264.7 macrophages with a GFP-labelled, constitutively active form of Rab1b [eGFP-Rab1b(CA)]. eGFP-Rab1b(CA) contains a mutation in the GTP binding domain that inhibits the hydrolysis of GTP, resulting in retention of the protein in the membrane in which the Rab GTPase is recruited [43, 46, 67, 68]. Twenty-four hours after transfection, macrophages were infected with either live or PFA killed *Y*. *pestis* CO92 pCD1^(-)^ pGEN::mCherry or *E*. *coli* K12 pGEN::mCherry, which constitutively express the mCherry fluorescent protein. Cells were washed and fixed with paraformaldehyde at 20 and 80 min post-infection and analyzed by confocal microscopy to determine localization of eGFP-Rab1b(CA) (Fig 6). Less than 25% of *E*. *coli* or PFA killed *Y*. *pestis*, which traffic to acidified vacuoles, colocalized with eGFP-Rab1b(CA) at 20 min post-infection (Fig 6B). Furthermore, we observed no significant change in colocalization at 80 min post-infection. However, in cells infected with live *Y*. *pestis*, we observed a significant increase in eGFP-Rab1b(CA) localization to the YCV at both time points (Fig 6B and 6C; ~57%; p≤0.05). These data demonstrate that while Rab1b is minimally associated with phagosomes containing *E*. *coli* or dead *Y*. *pestis*, the GTPase is associated with the YCV containing live *Y*. *pestis* at a significantly higher frequency, suggesting that Rab1b recruitment or retention to the YCV specifically contributes to *Y*. *pestis* survival.

### Disruption of the secretory pathway does not alter *Y*. *pestis* survival or inhibition of YCV acidification

**Fig 7:**
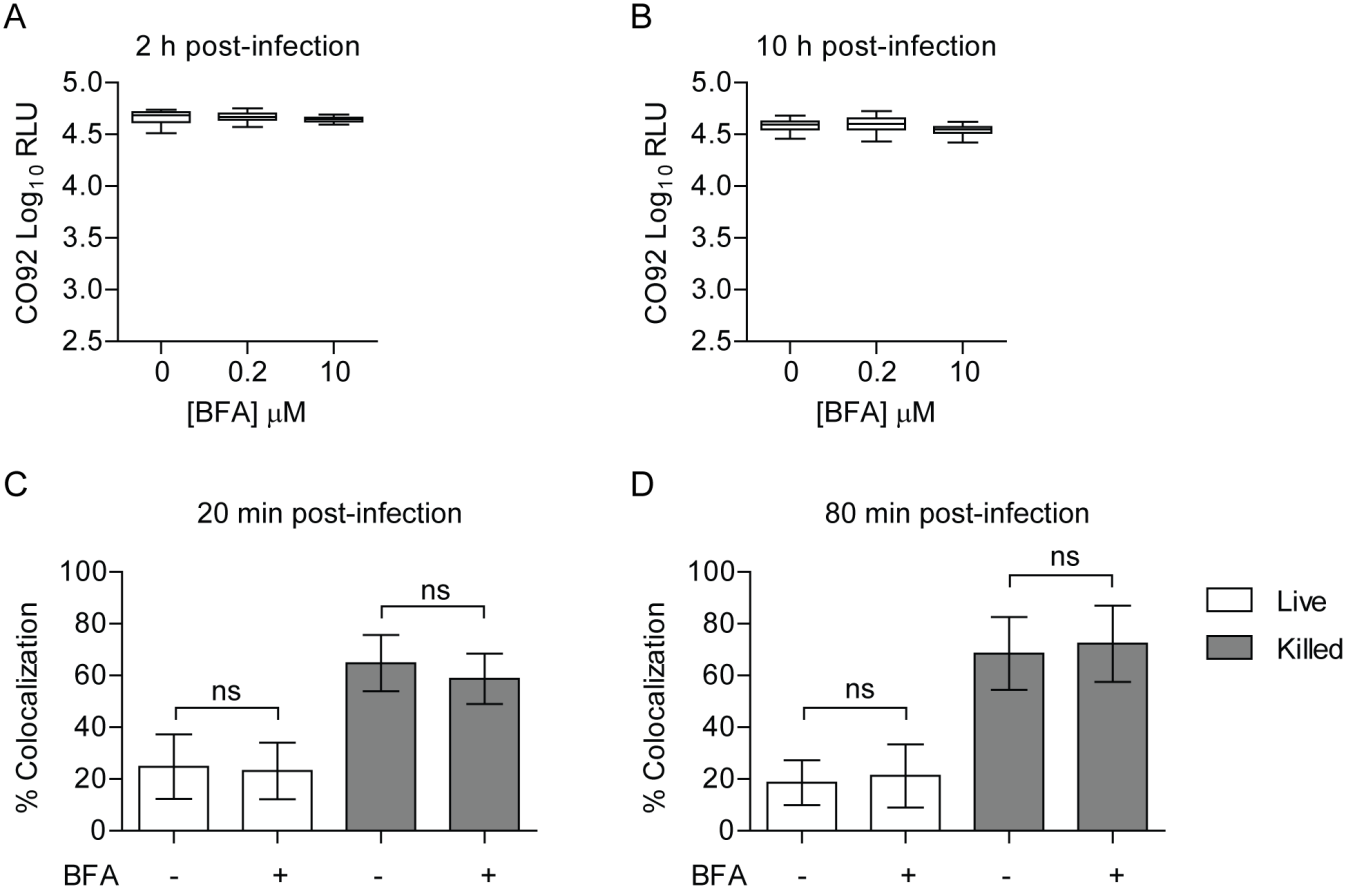
Inhibition of the secretory pathway does not inhibit *Y*. *pestis* intracellular survival. RAW264.7 macrophages were treated with 0, 0.2 or 10 μM BFA prior to infection with *Y*. *pestis* CO92 pCD1^(-)^ Lux_PtolC_ (MOI 10). Extracellular bacteria were killed with gentamicin and intracellular bacterial numbers were monitored at (A) 2 h and (B) 10 h post-infection by bioluminescence. To determine if BFA treatment impacted the ability of *Y*. *pestis* to inhibit YCV acidification, macrophages treated with 10 μM BFA were incubated with Lysotracker Red DND-99 prior to infection with live or paraformaldehyde killed *Y*. *pestis* CO92 pCD1^(-)^ pGEN222 (MOI 3). Bacterial Colocalization with Lysotracker Red DND-99 was determined by confocal microscopy at (C) 20 and (D) 80 min post-infection. ns = not significant.

Rab1b has an important role in mediating ER-to-Golgi trafficking [69, 70]. While Rab1b appears to be directly recruited to the YCV, it is also possible that the effect of Rab1b knockdown on *Y*. *pestis* survival is due to changes in Golgi trafficking. To determine if Golgi trafficking, specifically secretory trafficking, is required for *Y*. *pestis* to inhibit YCV acidification, we treated RAW264.7 macrophages with Brefeldin A (BFA), which blocks Golgi trafficking independent of Rab1b by targeting Arf1. BFA-treated macrophages were infected with *Y*. *pestis* CO92 pCD1^(-)^ Lux_PtolC_ for 20 min, extracellular bacteria were killed with gentamicin, and intracellular bacteria bioluminescence was monitored at 2 and 10 h post infection (Fig 7A and 7B, respectively). At both time points there was no significant difference in the survival of *Y*. *pestis* between untreated macrophages or cells treated with increasing concentrations of BFA. Macrophages treated with 10 μM BFA were also incubated with Lysotracker Red DND-99 and subsequently infected with *Y*. *pestis* CO92 pCD1^(-)^ pGEN222 to determine if inhibition of the secretory pathway altered YCV acidification. As a control, a separate group of cells were infected with paraformaldehyde killed *Y*. *pestis* CO92 pCD1^(-)^ pGEN222. In agreement with the intracellular bacterial survival, there was no significant difference between YCV acidification in BFA-treated macrophages at 20 or 80 min post-infection compared to untreated cells (Fig 7C and 7D). Furthermore, BFA treatment did not alter the acidification of phagosomes containing paraformaldehyde killed *Y*. *pestis*. Together these data demonstrate that *Y*. *pestis* avoidance of the phagolysosome is independent of retrograde endocytic trafficking and suggests that Rab1b impacts YCV maturation independent of its function in Golgi trafficking.

### Rab1b inhibition results in increased acidification of the Legionella containing vacuole

**Fig 8:**
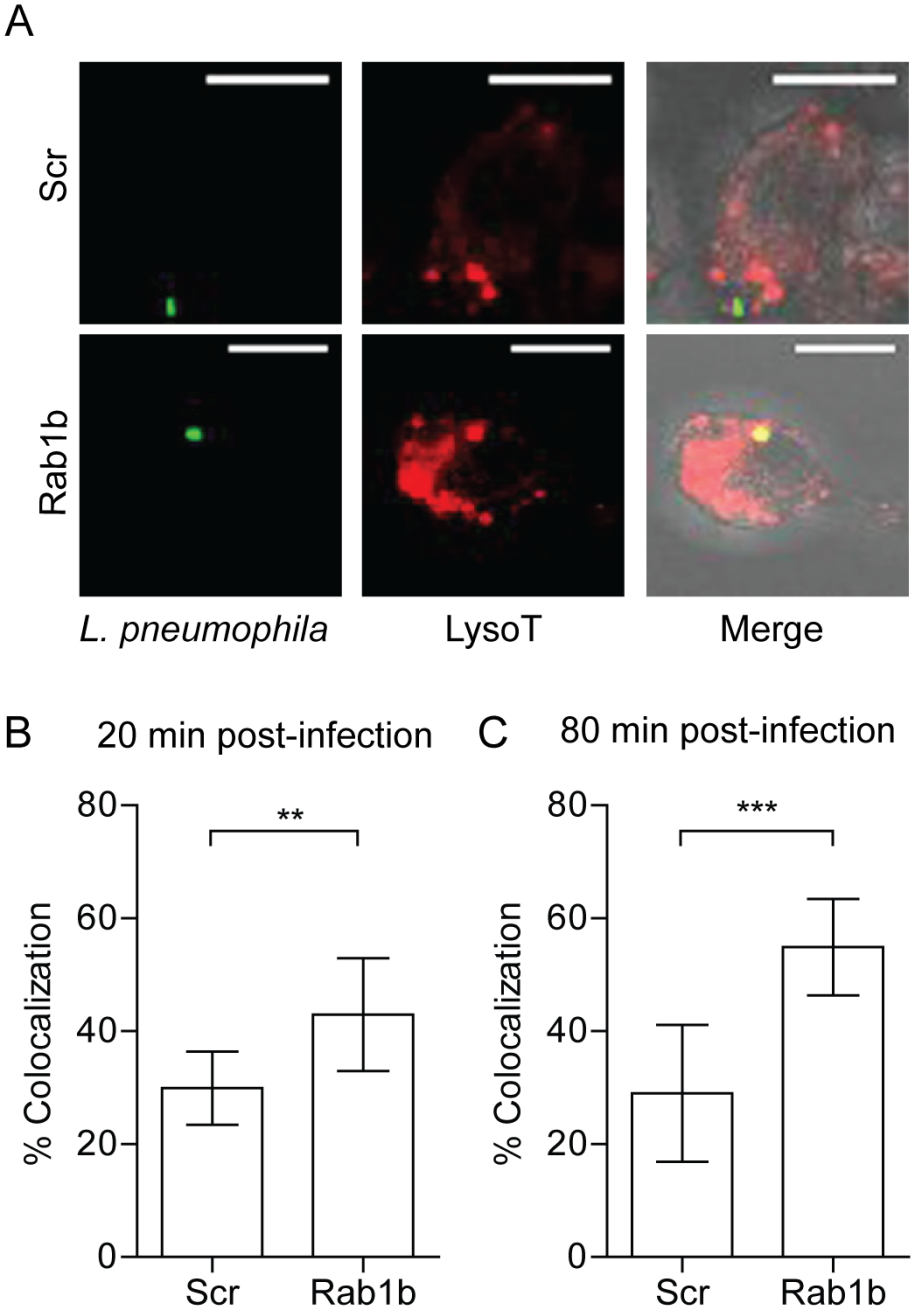
Knockdown of Rab1b increases *L*. *pneumophila* LCV acidification. RAW264.7 macrophage cells were reverse transfected with either scrambled (Scr) or Rab1b siRNA. 48 h after transfection cells were incubated with Lysotracker Red DND-99 for 1 h, and infected with *L*. *pneumophila* pMIP-GFP (MOI 10). Coverslips were fixed and colocalization of Lysotracker was determined by confocal microscopy. (A) Representative images showing colocalization of Lysotracker with *L*. *pneumophila*. Scale bar is 5μm. (B) Percent of LCVs that colocalized with Lysotracker at 20 min post-infection. (C) Percent of LCVs that colocalized with Lysotracker Red DND-99 at 80 min post-infection. ** = p<0.05, *** = p<0.001.

Previous studies with *L*. *pneumophila* demonstrate the cyclic recruitment and release of Rab1b on the LCV within 2 hours post-infection [45]. The release of Rab1b from the nascent LCV coincides with the transition of the LCV from a neutral to acidic pH [71, 72]. Given that *Y*. *pestis* recruits Rab1b to the YCV to prevent vacuole acidification, we hypothesized that *L*. *pneumophila* recruitment of Rab1b may also result in arrest of LCV acidification. To test this hypothesis, we transfected RAW264.7 macrophage cells with siRNA targeting Rab1b and treated transfected cells with Lysotracker Red DND-99 prior to infection with *L*. *pneumophila* to monitor LCV acidification. As previously reported, we observed that the majority of LCVs did not colocalize with Lysotracker in scramble siRNA treated macrophage (only 30% of *L*. *pneumophila* was found in acidified compartments by 80 min post-infection; Fig 8). In contrast, we observed a significant increase in Lysotracker colocalization in macrophages treated with siRNA targeting Rab1b at both 20 and 80 min post-infection (Fig 8B and 8C; p≤0.01 and P≤0.001, respectively). These data demonstrate that like *Y*. *pestis*, *L*. *pneumophila* requires Rab1b to inhibit LCV acidification during early stages of macrophage infection.

## Discussion

Rab proteins are central mediators in vesicular trafficking within the cell. As such, intracellular pathogens often target these GTPases to subvert the normal phagosome maturation pathway and survive within host cells (see [25, 73] for reviews). Rab1 was one of the first identified members of this family and has been extensively studied for its role in Golgi trafficking in yeast, *Drosophila*, and mammalian cells (see [70, 74, 75] for reviews). More recently, both isoforms of Rab1 have been linked to intracellular infection by several pathogens. *Chlamydial* species [48], *L*. *pneumophila* [40, 46], *A*. *phagocytophilum* [43], *Coxiella burnetii* [39], and *S*. *enterica Typhimurium* [44] have been shown to recruit Rab1 to the PCV. Furthermore, inhibition of Rab1 by either RNAi or expression of dominant negative Rab1 constructs indicate that Rab1 function is required for the survival/growth of *L*. *pneumophila* [40], *C*. *burnetii* [39], *S*. *enterica Typhimurium* [44, 76], and *Brucella melitensis* [77]. Our data demonstrate for the first time that *Y*. *pestis* also belongs to this group. Specifically, we have demonstrated that *Y*. *pestis* recruits Rab1b to the YCV during infection of macrophages and that this GTPase is required for intracellular survival. Interestingly, Rab1 has only been shown to be required for the survival of pathogens that exist within vacuolar compartments, suggesting a role(s) for Rab1 in subverting normal phagosome maturation and generation of a protective PCV. In fact, functional Rab1 has been shown to be detrimental to the survival of the cytoplasmic pathogen *Shigella flexnerii* through its interaction with the autophagy system within the host cell [41]. However, *S*. *flexnerii* has also evolved to target Rab1, through the VirG secreted effector protein, and inactivate the GTPase to inhibit macroautophagy during infection [41].

While Rab1 has been linked to the survival of several intracellular pathogens, the role Rab1 plays in the maturation of individual PCVs is less well understood. In *C*. *burnetii*, Rab1 has been shown to be required for the massive expansion of the *Coxiella* replicative vacuole (CRV) [39]. This requires the acquisition of new membrane in order for the CRV to grow, and Rab1 recruitment to the vacuole may mediate the interception of vesicles (and their membranes) from the secretory pathway. This hypothesis is supported by studies showing that treatment with BFA, which independently inhibits the secretory pathway, also inhibits the expansion of the CRV [39]. Studies from *A*. *phagocytophilum* and Chlamydial species, which also form a large replicative vacuole, also suggest that Rab1 recruitment is important for formation of a spacious vacuolar compartment [43, 48]. Therefore, a common goal of bacteria that recruit Rab1 to their PCV may be to subvert the secretory pathway in order to remodel the PCV. Furthermore, Rab1b has also been linked to autophagy [44], which is also associated with the replication of both *C*. *burnetii* and *A*. *phagocytophilum* [78, 79]. It is possible that in addition to the secretory pathway, Rab1 recruitment may also contribute to the recruitment of autophagsomal membranes to these PCV, though this has yet to be demonstrated. Since the YCV also expands late during infection (though not to the degree of these former pathogens) to form a spacious vacuole [5, 12, 18], it is possible that Rab1b may contribute to YCV expansion. However, we have not observed changes in spacious YCV formation in Rab1b siRNA treated macrophages. Furthermore, our data also suggest that early association with the autophagosome marker LC3 does not appear to protect YCV from acidification, as we observed no difference in YCV-LC3 association in Rab1b siRNA treated cells. More importantly, our data with *Y*. *pestis* reveal a potential new benefit of Rab1 recruitment to the PCV, which is to avoid phagosomal acidification and subsequent fusion to the lysosome. While it is currently unclear how Rab1b inhibits YCV acidification, it appears to be independent from its contributions to the secretory pathway, as BFA treatment did not result in similar changes to YCV acidification. Importantly, while knockdown of Rab1B does not alter the expression of Rab 5, 7 or 9, which are required for phagosome maturation (S1A Fig), it is possible that recruitment and retention of Rab1b to the early phagosome inhibits interactions with these Rabs (and/or Rab effector proteins) to inhibit normal phagosome maturation. Rab1 has also been linked to endosomal sorting through direct interactions with the kinesin Kifc1, which in turn affects directional vesicular motility within the cell [55, 56]. Thus, Rab1b recruitment may alter early sorting of the YCV to avoid acidification and lysosomal fusion. Studies to better characterize the early YCV, including differences in Rab composition and vATPase recruitment as compared to the normal phagosome are ongoing and will provide further insight into these mechanisms. Rab1 recruitment to the YCV also occurs significantly earlier than reported for *C*. *burnetii* (≤20 min vs. >12 h, respectively) [39], suggesting that timing of recruitment may indicate which function, inhibition of phagosome maturation or membrane acquisition, is contributing to pathogenesis of various pathogens. It should be noted that *C*. *burnetii* requires passage through an acidified vacuole to induce the expression of important virulence factors and subsequent intracellular survival [80]. Therefore, our observations that early acquisition of Rab1 inhibits PCV acidification may explain why Rab1 recruitment is delayed during C. *burnetii* infection. In contrast to *C*. *burnetii*, *L*. *pneumophila*, which inhibits LCV acidification early during infection [71, 72, 81], recruits Rab1 in a similar time frame as seen during *Y*. *pestis* infection (within 10 min) [46]. In support of our hypothesis that early recruitment of Rab1b is a mechanism for pathogens to inhibit phagosome acidification, we demonstrated that knockdown of Rab1b decreased the ability of *L*. *pneumophila* to inhibit LCV acidification (Fig 8). Interestingly, *L*. *pneumophila* appears to control both recruitment and later release of Rab1 from the LCV (discussed below). The timing of Rab1 modification by *L*. *pneumophila* coincides with a transition from a neutral to an acidic LCV [71, 72], suggesting that Rab1 inhibition of acidification may be an active process that is reversible upon removal of Rab1 from the vacuolar membrane.

Phagosome acidification has been shown to be a key step in phagosome maturation. Acidification of the phagosome is believed to work in concert with Rab5, Rab7 and Rab9 to mediate phagosome maturation and ultimately fusion with lysosomes [27, 28]. Initially, the early phagosome, highlighted by association with Rab5, is slightly acidic (~pH 6.0). As the phagosome matures, the pH decreases and Rab7 replaces Rab5 on the phagosome. Rab7 subsequently recruits more vATPase complexes, resulting in further acidification of the phagosome and recruitment of Rab9. By the time Rab9 mediates lysosomal fusion, the pH of the phagosome is approaching 4.0, which is the optimal pH to activate hydrolases and proteases delivered to the phagosome by the lysosome. Several lines of evidence indicate that acidification of the phagosome is required in order for efficient lysosomal fusion and function to occur [31, 32, 82–84], which suggest that inhibition of acidification could influence proper lysosomal fusion to the PCV. In line with these hypotheses, we observed a direct correlation between increased YCV acidification with increased Lamp1 association, and subsequent decreased *Y*. *pestis* survival, in Rab1b siRNA treated cells. This direct correlation makes it difficult to separate the impact of acidification directly on *Y*. *pestis* survival (acidic killing) from lysosomal fusion, but further supports the importance of inhibiting YCV acidification as mechanism for *Y*. *pestis* intracellular survival [12].

While Rab1 is important for the intracellular survival of several pathogens, bacterial virulence factors that target Rab1 have only been identified for *Chlamydia* [48] and *L*. *pneumophila* [45, 47, 85–91]. In the case of *L*. *pneumophila*, multiple Dot/Icm secreted factors have been shown to target Rab1 and modify the protein to manipulate localization to the LCV; cycling the host Rab1 between active (anchored to the LCV) and inactive states. The effectors DrrA/SidM, SidD and LepB work in concert to first recruit Rab1 to LCV, and then later remove it [42, 47, 59, 62, 92]. *L*. *pneumophila* also manipulates Rab1 independent of recruitment to the LCV through the action of SidC/SdcA, LidA and AnkX [85, 89, 91, 93]. The redundancy in Rab1 targeting proteins indicates that Rab1 manipulation by *L*. *pneumophila* is extremely important for the intracellular survival of this pathogen. For *Y*. *pestis*, we have yet to define the virulence factors that mediate Rab1b recruitment to the YCV. However, we have shown that *Y*. *pestis* does not require the pCD1 plasmid (including the Ysc T3SS) or the high pathogenicity island (pgm locus) to recruit Rab1b and inhibit YCV acidification. These findings are in agreement with previous work that has shown both of these genetic elements are dispensable for intracellular survival [5, 8, 14, 20]. Therefore, virulence factors encoded elsewhere in the genome are mediating both Rab1b interactions and intracellular survival. While the PhoPQ two component regulator has been shown to contribute to intracellular survival, likely through the regulation of other genes [13, 15, 22, 23], we speculate that these genes do not regulate survival through Rab1b because *phoPQ* mutants still inhibit YCV acidification during infection [13]. However, defining Rab1b recruitment to the *phoPQ* mutant YCV is needed to confirm this hypothesis. Studies to specifically identify *Y*. *pestis* factors involved in Rab1b recruitment to the YCV are ongoing.

In summary, we have shown here for the first time that recruitment of Rab1b to the PCV directly correlates to the ability of a pathogen to inhibit acidification of the vacuole. These findings indicate a novel function for Rab1b in inhibiting phagosome maturation and suggest that other pathogens may use a similar strategy to modify the maturation of the PCV. Furthermore, in the context of *Y*. *pestis* infection, Rab1b is the first factor, either host or bacterial, identified that directly impacts acidification of the YCV. Future studies to define how Rab1b impacts phagosome acidification and to identify additional host factors that contribute YCV biogenesis will be important for us to understand how this pathogen evades killing by macrophages.

## Materials and Methods

### Bacterial strains, plasmids, and macrophages

All bacterial strains used in this study are listed in S1 Table in the Supporting Information. *Y*. *pestis* CO92 [94] pCD1^(-)^ and KIM D-19 (pgm^(-)^) (BEI Resources) were cultivated at 26°C in Brain Heart Infusion (BHI) broth (Difco). When needed, carbenicillin was used at 50μg/mL. Bioluminescent derivatives were generated using the Lux_PtolC_ bioreporter as described previously [63]. To generate fluorescent bacterial strains, *Y*. *pestis* and *E*. *coli* K12 DH5α were transformed with pGEN222, pGEN-*P*_*EM7*_::DsRED, or pGEN222::mCherry [66]. *E*. *coli* was cultivated at 37°C in Luria-Bertani (LB) broth (Difco) supplemented with 50μg/mL carbenicillin. *L*. *pneumophila* AA100, a clinical isolate containing pMIP-GFP, was grown on BCYE agar plates for 3 days at 37°C prior to macrophage infection [95–97]. The pGEN222::mCherry plasmid was generated by replacing the EGFP gene from pGEN222 with the mCherry gene using Gibson Cloning [98]. Constitutive active EGFP-Rab1b was generated by site directed mutagenesis of pEGFP-Rab1b [99] using primers 5’- TGG AAC GGT TCC GGA C -3’ and 5’- GGC CCG CTG TGT CC -3’ to mutate the Glutamine at residue 67 to a Leucine as previously described [99]. RAW264.7 macrophages were obtained from ATCC and cultured in DMEM, 100 mM glucose + 10% FBS (Hyclone).

### Transfection of macrophages

For siRNA transfection, 20 μl of 0.165 μM Silencer siRNA (Life Technologies) diluted in Opti-MEM (Life Technologies) was mixed with 10 μl of 0.03% (v/v) Lipofectamine RNAiMax/Opti-MEM (Life Technologies) as described by the manufacturer. 30 μl of the siRNA-Lipofectamine complex was added to each well of a white flat-bottom 96-well plate (Greiner), incubated at room temperature for 10 min, and then 1x10^4^ RAW264.7 macrophages suspended in 80 μl of DMEM+10% FBS were added. Cells were incubated for 48 h at 37°C with 5% CO_2_. For 24-well plates used for microscopy, all reagents were increased by 4-fold. For plasmid transfection, 4 μg of plasmid was transfected into 4.4 x 10^5^ RAW264.7 macrophages using Lipofectamine 2000 (Life Technologies) or 0.5 μg of plasmid with JetPrime (Polyplus) as described by the manufacturers. Luminescence was monitored with a Synergy 4 plate reader (BioTek) (1 sec read with sensitivity set at 150).

### Bacterial infection of macrophages

Macrophages were infected with *Y*. *pestis* strains as previously described [11, 63]. Briefly, bacteria were grown at 26°C in BHI, washed in PBS, and diluted appropriately in prewarmed DMEM+10%FBS. Bacteria were added to macrophages and the infection was synchronized by centrifugation. After 20 min, extracellular bacteria were killed with gentamicin (16μg/mL). One hour after gentamicin treatment, the medium was replaced with DMEM + 10% FBS containing 2μg/mL gentamicin. Intracellular *Y*. *pestis* numbers were determined by bioluminescence using a Synergy HT plate reader (Biotek) or conventional bacterial enumeration as described previously [63]. For *L*. *pneumophila*, bacteria were swabbed directly from plates and diluted appropriately in prewarmed DMEM+10%FBS. Bacteria were added to macrophages and the infection was synchronized by centrifugation. At 20 minutes and 80 minutes post-infection cell monolayers were washed three times with PBS and fixed as described below [96, 97]. All MOIs were confirmed by conventional enumeration of the inoculum at the time of infection. For vacuole acidification experiments, 75 nM Lysotracker Red DND-99 (Life Technologies) was added to the cells 1 h prior to fixation. Brefeldin A (Sigma) was added to cells 2 h prior to *Y*. *pestis* infection and maintained throughout the infection.

### Immunofluorescent staining and confocal microscopy

For confocal microscopy, cells were fixed to coverslips with 4% paraformaldehyde for 30 min. For indirect immunofluorescent staining, fixed cells were blocked with 3% BSA overnight and incubated with rabbit anti-*Y*. *pestis* serum (1:1,000), anti-Lamp1 (0.8ug/ul; Abcam ab24170), or anti-MAP-LC3α/β (1:200; Santa Cruz sc-16756) antibodies for 1 h. Unbound primary antibodies were removed by washing and anti-rabbit Alexa Fluor 488 secondary antibody (1:4000; Life Technologies) was added for 1 h. All coverslips were mounted with Prolong Gold with DAPI (Life Technologies) and imaged on an Olympus FV100 laser or Zeiss LSM 710 laser confocal microscope. Colocalization of Lysotracker Red DND-99 or proteins to the YCV was determined using the Coloc function in the Imaris image analysis software (BitPlane).

### Statistics

All data are shown as mean and standard deviation of three to six biological replicates and each experiment was repeated three times to confirm the phenotypes. For microscopy, at least 50 vacuoles per biological replicate were analyzed. p-values were calculated by one-way ANOVA (or t-test for *L*. *pneumophila* experiments) using GraphPad Prism software.

## Supporting Information

S1 Fig Transfection with Rab1b siRNA in macrophages. RAW264.7 macrophages were reverse transfected with either scrambled (Scr) or Rab1b siRNA and incubated for 48 h. (A) Total RNA was isolated from transfected cells (n = 5) and Rab1b, Rab1a, Rab5a, Rab7 and Rab9 transcript levels were determined by qRT-PCR (Rab1b primers: 5’-TGTCCTTTGTGCTGTCTCTTG -3’ and 5’- TCATCCTTTTCCATCTTCCCC -3’; Rab1a primers: 5’- CCTGCCTTCTCCTTAGGTTTG -3’ and 5’- TCGAAATCTTTCCTGGCCTG -3’; Rab5a primers: 5’- TGGTCAAGAACGGTATCATAGC -3’ and 5’- GCCTTTGAAGTTCTTTAACCCAG -3’; Rab7 primers: 5’- AATAGGAGCGGACTTTCTGAC -3’ and 5’- CATCAAACACCAGAACACAGC -3’); Rab9 primers: 5’- CACGGAAGATAGGTCAGAACAC -3’ and 5’- CCCTTTAATGCCATCAACAGC -3’); GapDH primers: 5’- AATGGTGAAGGTCGGTGTG -3’ and 5’- ACAAGCTTCCCATTCTCGG -3’). Relative expression was calculated using the ΔΔCt method [100]. Only Rab1b levels were significantly altered in Rab1b siRNA-treated cells compared to scramble treated cells (** = p<0.01; Student’s T-test). (B) Whole cell lysates were harvested from transfected cells and Rab1b protein levels (anti-Rab1b(G-20); Santa Cruz sc-599) were determined by Western blot. β-actin (anti-β-Actin; Abcam ab8227) represents loading control. (C) Cell viability of Rab1b siRNA transfected cells was determined using Cell Titer-Glo as described by the manufacturer (Promega). No significant difference in viability was observed between Rab1b siRNA treated cells and scramble siRNA-treated (Scr), untransfected macrophages (Cells), or macrophages treated with Lipofectamine without siRNA (Lipo). RLU = Relative Light Units.
(TIF)

S2 Fig Growth at 37°C does not alter intracellular survival of *Y*. *pestis*. RAW264.7 macrophages were reverse transfected with Rab1b, scrambled (Scr), or Copβ1 siRNA. 48 h after transfection cells were infected with *Y*. *pestis* CO92 pCD1^(-)^ Lux_PtolC_ (MOI 10) grown for 3 h at 37°C prior to infection. (A) Bioluminescence of intracellular bacteria from macrophages infected for 2 h. (B) Bioluminescence of intracellular bacteria from macrophages infected for 10 h. RLU = relative light units. *** = p<0.001.
(TIF)

S3 Fig Rab1b knockdown inhibits the survival of enteric *Yersinia* within macrophages. RAW264.7 macrophages were reverse transfected with Rab1b, scrambled (Scr), or Copβ1 siRNA. 48 h after transfection cells were infected with pYV cured *Y*. *enterocolitica* 8081 [101] or *Y*. *pseudotuberculosis* IP32952 [102](MOI 10). Extracellular bacteria were killed with gentamicin and at 2 and 10 h post infection intracellular bacteria were determined by conventional enumeration. (A) Intracellular *Y*. *enterocolitica* at 2 h post infection. (B) Intracellular *Y*. *enterocolitica* at 10 h post infection. (C) Intracellular *Y*. *pseudotuberculosis* at 2 h post-infection (D) Intracellular *Y*. *pseudotuberculosis* at 10 h post-infection. The limit of detection for conventional enumeration is 2.5 log_10_ CFU. CFU = colony forming units. * = p<0.05, *** = p<0.001.
(TIF)

S1 Table Bacterial strains used in these studies. (DOCX)
