# Supplementary figures and images for: Yersinia pestis Requires Host Rab1b for Survival in Macrophages
Source: PLoS Pathog. 2015 Oct 23;11(10):e1005241. doi: 10.1371/journal.ppat.1005241 (PMC4619670; doi:10.1371/journal.ppat.1005241)

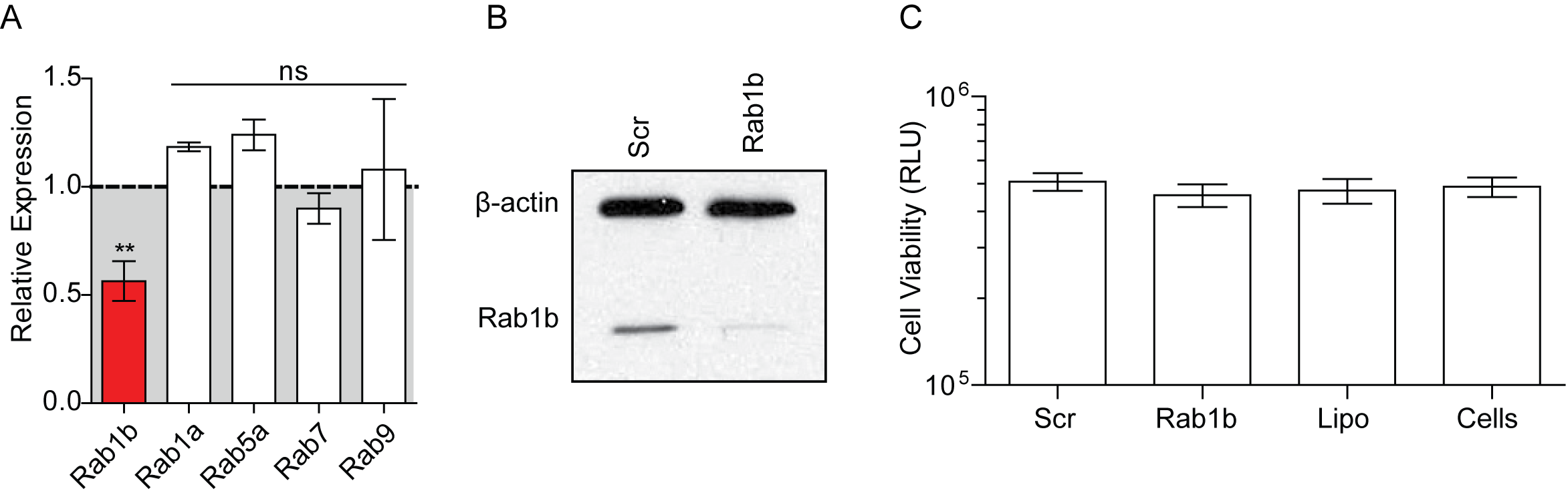

Supplement: S1 Fig — RAW264.7 macrophages were reverse transfected with either scrambled (Scr) or Rab1b siRNA and incubated for 48 h. (A) Total RNA was isolated from transfected cells (n = 5) and Rab1b, Rab1a, Rab5a, Rab7 and Rab9 transcript levels were determined by qRT-PCR (Rab1b primers: 5’-TGTCCTTTGTGCTGTCTCTTG -3’ and 5’- TCATCCTTTTCCATCTTCCCC -3’; Rab1a primers: 5’- CCTGCCTTCTCCTTAGGTTTG -3’ and 5’- TCGAAATCTTTCCTGGCCTG -3’; Rab5a primers: 5’- TGGTCAAGAACGGTATCATAGC -3’ and 5’- GCCTTTGAAGTTCTTTAACCCAG -3’; Rab7 primers: 5’- AATAGGAGCGGACTTTCTGAC -3’ and 5’- CATCAAACACCAGAACACAGC -3’); Rab9 primers: 5’- CACGGAAGATAGGTCAGAACAC -3’ and 5’- CCCTTTAATGCCATCAACAGC -3’); GapDH primers: 5’- AATGGTGAAGGTCGGTGTG -3’ and 5’- ACAAGCTTCCCATTCTCGG -3’). Relative expression was calculated using the ΔΔCt method [100]. Only Rab1b levels were significantly altered in Rab1b siRNA-treated cells compared to scramble treated cells (** = p<0.01; Student’s T-test). (B) Whole cell lysates were harvested from transfected cells and Rab1b protein levels (anti-Rab1b(G-20); Santa Cruz sc-599) were determined by Western blot. β-actin (anti-β-Actin; Abcam ab8227) represents loading control. (C) Cell viability of Rab1b siRNA transfected cells was determined using Cell Titer-Glo as described by the manufacturer (Promega). No significant difference in viability was observed between Rab1b siRNA treated cells and scramble siRNA-treated (Scr), untransfected macrophages (Cells), or macrophages treated with Lipofectamine without siRNA (Lipo). RLU = Relative Light Units. (TIF) [file ppat.1005241.s001.tif]

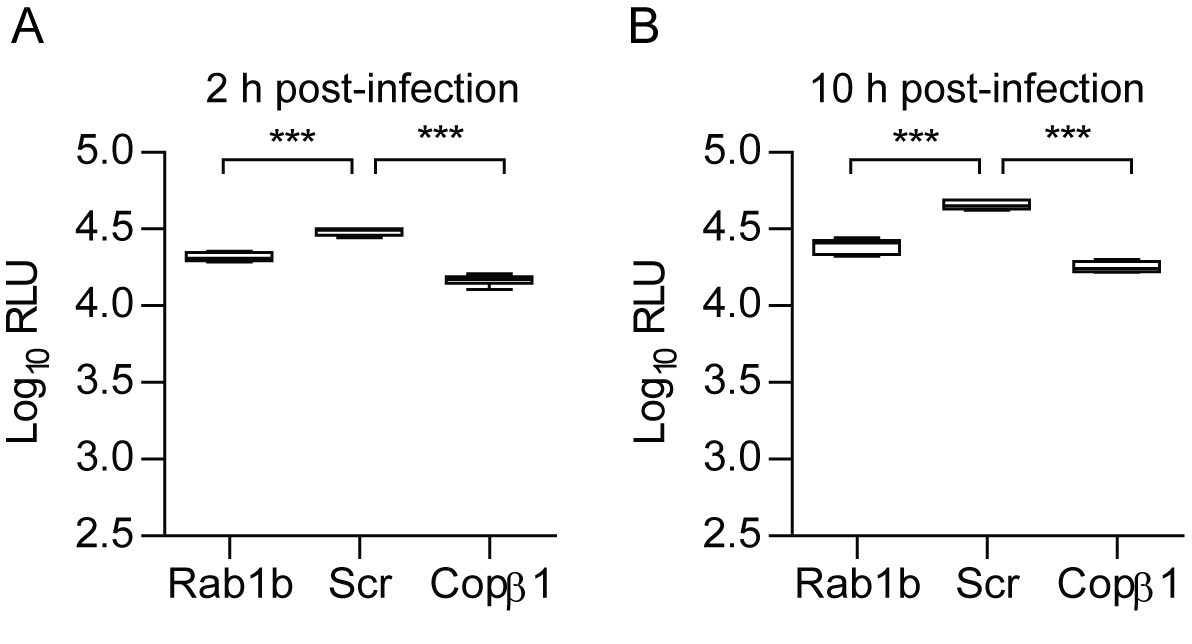

Supplement: S2 Fig — RAW264.7 macrophages were reverse transfected with Rab1b, scrambled (Scr), or Copβ1 siRNA. 48 h after transfection cells were infected with Y. pestis CO92 pCD1(-) LuxPtolC (MOI 10) grown for 3 h at 37°C prior to infection. (A) Bioluminescence of intracellular bacteria from macrophages infected for 2 h. (B) Bioluminescence of intracellular bacteria from macrophages infected for 10 h. RLU = relative light units. *** = p<0.001. (TIF) [file ppat.1005241.s002.tif]

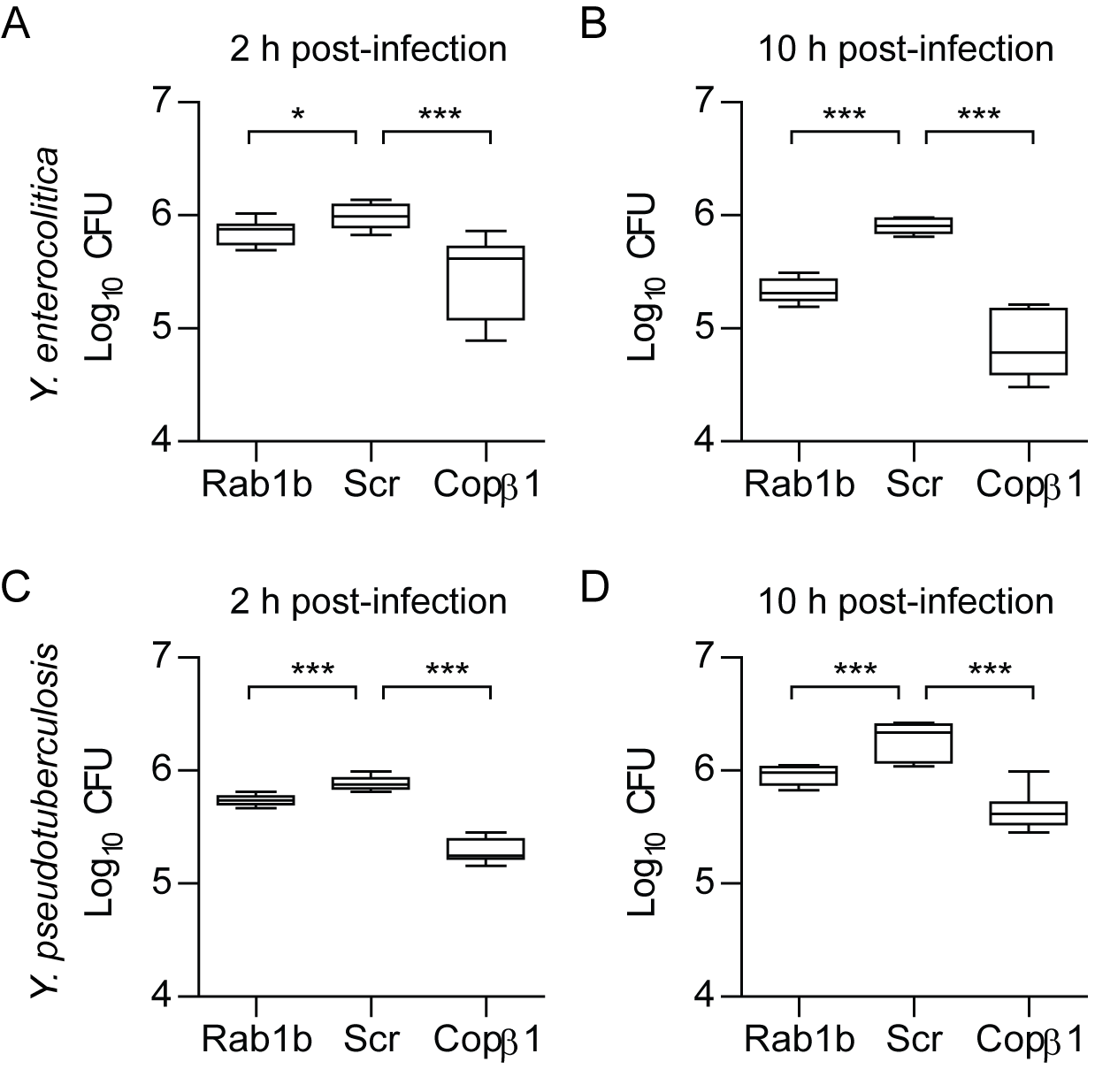

Supplement: S3 Fig — RAW264.7 macrophages were reverse transfected with Rab1b, scrambled (Scr), or Copβ1 siRNA. 48 h after transfection cells were infected with pYV cured Y. enterocolitica 8081 [101] or Y. pseudotuberculosis IP32952 [102](MOI 10). Extracellular bacteria were killed with gentamicin and at 2 and 10 h post infection intracellular bacteria were determined by conventional enumeration. (A) Intracellular Y. enterocolitica at 2 h post infection. (B) Intracellular Y. enterocolitica at 10 h post infection. (C) Intracellular Y. pseudotuberculosis at 2 h post-infection (D) Intracellular Y. pseudotuberculosis at 10 h post-infection. The limit of detection for conventional enumeration is 2.5 log10 CFU. CFU = colony forming units. * = p<0.05, *** = p<0.001. (TIF) [file ppat.1005241.s003.tif]
