# Supplementary material for: Yersinia pestis Requires Host Rab1b for Survival in Macrophages
Source: PLoS Pathog. 2015 Oct 23;11(10):e1005241. doi: 10.1371/journal.ppat.1005241 (PMC4619670; doi:10.1371/journal.ppat.1005241)
Supplement: S1 Table — (DOCX) [file ppat.1005241.s004.docx]

| **Table S1. Bacterial Strains** |  |  |
| --- | --- | --- |
| **Bacterial Strains** |  | **References/sources** |
| *Y. pestis* CO92 pCD1^(-)^ |  | [[94](#_ENREF_94)] |
| *Y. pestis* KIM D-19 pgm^(-)^ pCD1^(+)^ |  | BEI Resources |
| *Y. pseudotuberculosis* IP32593 pYV^(-)^ |  | [[102](#_ENREF_102)] |
| *Y. enterocolitica* 8081 pYV^(-)^ |  | [[101](#_ENREF_101)] |
| *E. coli* DH5α |  | New England Biolabs |
| *L. pneumophila* AA100 |  | [[95](#_ENREF_95)] |
